# Symptom networks of therapy-seeking individuals with comorbid psychosis and PTSD

**DOI:** 10.1017/S0033291726104978

**Published:** 2026-07-28

**Authors:** Rachel Frost, Ciarán O’Driscoll, Emmanuelle Peters, Filippo Varese, Craig Steel, Raphael Underwood, Sarah Swan, Amy Hardy

**Affiliations:** 1 Institute of Psychiatry Psychology & Neuroscience, King’s College London, UK; 2 University College London, UK; 3 South London and Maudsley NHS Foundation Trust, UK; 4 University of Manchester, UK; 5 University of Oxford, UK

**Keywords:** complex PTSD, delusions, hallucinations, posttraumatic cognitions, psychosis, post-traumatic stress, trauma

## Abstract

**Background:**

Post-traumatic sequelae are theorized to contribute to and maintain psychotic symptoms. The relative contribution of core posttraumatic stress disorder (PTSD) symptoms and those of complex PTSD are understudied. Network analysis was used to examine interactions between psychosis, core PTSD, and complex post-traumatic symptoms. Based on previous evidence, it was predicted that trauma-related beliefs would have the greatest predictability. It was also hypothesized that the association between voices and trauma-related beliefs would be greater than the association between voices and re-experiencing.

**Methods:**

Baseline data from the Study of Trauma and Recovery (STAR), a randomized controlled therapy trial of people with schizophrenia-spectrum disorder who experience distressing delusions and/or hallucinations and comorbid PTSD (*N* = 305). A network was estimated using a Bayesian Gaussian graphical model, comprised of psychosis symptoms (delusions and voices), posttraumatic stress symptoms (re-experiencing, avoidance, and sense of threat), complex posttraumatic stress symptoms (affective dysregulation, negative self-concept, disturbed relationships), and trauma-related beliefs (negative cognitions about the self and world, self-blame). A direct acyclic graph was plotted to estimate the potential directionality of associations between symptoms.

**Results:**

The hypotheses were not supported. All symptoms had similar predictability scores. There was no evidence for conditionally dependent relations between re-experiencing and voices nor between trauma-related beliefs and voices. However, self-blame was associated with delusions, and affective dysregulation was found to predict voices.

**Conclusions:**

Affective dysregulation and self-blame may maintain voices and delusions, respectively. Heterogeneity in trauma-related psychosis could explain inconsistent findings; future research should identify symptom presentation subgroups to improve network reliability.

## Background

Among individuals with a psychotic disorder, the presence of trauma and posttraumatic stress disorder (PTSD) is associated with worse functional impairment, reduced quality of life, symptom persistence, and poorer treatment outcomes (Hassan & De Luca, 2015; Seow et al., 2016; Thomas, Höfler, Schäfer, & Trautmann, 2019). A greater understanding of the relationship between posttraumatic sequelae and psychosis symptoms is warranted, as this may contribute to improved assessment and intervention (Hardy, van de Giessen, & van den Berg, 2020; Schäfer & Fisher, 2011).

In the context of a biopsychosocial vulnerability to psychosis, trauma may contribute to psychosis symptoms via trauma-related processes implicated in the development of PTSD such as emotion regulation, intrusive trauma memories, and cognitions or schemas (e.g. Berry & Bucci, 2016; Freeman et al., 2002; Garety et al., 2001; Hardy, 2017; Longden, Madill, & Waterman, 2012). For example, experiencing trauma may lead to attempts to manage the associated threat through hyperarousal or dissociation (Read, Fosse, Moskowitz, & Perry, 2014; Schäfer & Fisher, 2011). During a traumatic event, arousal-induced changes to information processing may result in decontextualized memories later emerging as intrusive trauma memories (Brewin & Patel, 2010). Poorly integrated intrusive trauma memories may then manifest as hallucinatory experiences, with their content directly or indirectly reflecting traumatic events (Berry & Bucci, 2016; Longden, Madill, & Waterman, 2012; McCarthy-Jones & Longden, 2015; van den Berg et al., 2023). Trauma memories shape hallucinatory content, and how these experiences are interpreted, potentially giving rise to paranoia. When individuals try to manage these threatening perceptions through avoidance, dissociation, or hypervigilance, they inadvertently reinforce their psychotic symptoms instead of reducing them (Freeman et al., 2002; Freeman & Garety, 2014).

Emotion regulation and negative beliefs have been shown to mediate the relationship between trauma and psychosis (Hardy et al., 2016; Peach et al., 2019; Williams, Bucci, Berry, & Varese, 2018), while evidence for intrusive trauma memories is inconclusive (Williams, Bucci, Berry, & Varese, 2018). Further, there has been relatively little research examining the relative contribution of core PTSD symptoms (re-experiencing, hyperarousal, and avoidance) and those reflective of complex PTSD (cPTSD), operationalized as ‘disturbances of self-disorganization’ (DSO) (negative self-concept, relationship difficulties, and affective dysregulation) (International Classification of Diseases 11th edition (ICD-11), World Health Organization (WHO, 2019). In a preliminary investigation, Panayi et al. (2022) found that both core PTSD and DSO symptoms mediated the relationship between trauma and positive symptoms in people with psychosis diagnoses, with PTSD having twice the effect compared to DSO. In a subsequent study using Ecological Sampling Methodology (ESM) in a sample of people with psychosis and PTSD, both DSO and PTSD were found to predict voices, visions, and paranoia, with the former found to have a larger effect in the flow of daily life, particularly for paranoia (Panayi et al., 2024). However, neither of these studies examined the relationship of specific DSO symptom clusters with psychosis.

To address this, network theory may offer further insight into the relationship between specific posttraumatic sequelae and psychosis symptoms. Rather than viewing symptoms as a passive consequence and equally interchangeable indicators of a disease entity or latent disorder, network theory assumes mental health difficulties arise from and are constitutive of a system of causally interrelated symptoms (Borsboom, 2017). The network perspective does not assume symptoms are interchangeable; certain symptoms may have a stronger causal influence compared to others, making it possible to identify the importance or centrality of symptoms within a network (Borsboom & Cramer, 2013). As the ‘disorder’ is an emergent property of these interactions, modelling symptoms can facilitate a transdiagnostic approach to examining relations amongst symptoms of different disorders (Cramer, Waldorp, van der Maas, & Borsboom, 2010).

Research employing network analysis to examine the relationship between PTSD and psychosis symptoms is emerging (Astill Wright et al., 2023; Hardy, O’Driscoll, Steel, van der Gaag, & van den Berg, 2021; Jin et al., 2022; Panayi et al., 2025). Hardy et al. (2021) examined the interplay between PTSD symptoms, psychosis symptoms, and posttraumatic beliefs among a clinical sample with comorbid PTSD and psychosis symptoms. The results indicated that trauma-related beliefs were the most central symptoms in the network. Voices were not directly associated with intrusive trauma memories, rather voice hearing and intrusive trauma memories were linked via trauma-related beliefs and delusions. This suggests that threat beliefs may play a stronger role in maintaining voices, compared to other mechanisms implicated in the literature, such as episodic trauma memories (Fowler et al., 2006; Freeman et al., 2002; Freeman & Garety, 2014; Garety et al., 2001; McCarthy-Jones & Longden, 2015). Panayi et al. (2025) extended these findings, by conducting a network study using an ESM study of participants with comorbid PTSD and psychosis to investigate the temporal dynamics of relationships between symptoms of PTSD, DSO, and psychosis in the flow of daily life. The results indicated that symptoms of PTSD, DSO, and psychosis may reinforce each other, especially paranoia with hyperarousal, negative self-concept, and emotional dysregulation. DSO symptoms did not predict voices temporally, which were instead predicted by paranoia.

The present study built on these findings to investigate associations between trauma-related beliefs, posttraumatic stress, and psychosis symptoms (i.e. voices and delusions) within a clinical sample with PTSD and psychosis using standardized assessments. The ICD-11 conceptualization of PTSD and cPTSD was adopted (WHO, 2019), enabling investigation of the relative contributions of core PTSD symptoms as well as DSO characteristic of cPTSD (WHO, 2019).

A novel aspect of this study is the use of Bayesian methods to test hypotheses within the network (Williams, 2021; Williams & Mulder, 2020). With network analytic approaches, attention needs to be given to estimation and interpretation. Network analysis generally adopts a data-driven exploratory approach, which may undermine the generalizability of findings across different samples (Fried et al., 2018). In contrast, Bayesian methodology has been introduced that can permit assessing the amount of evidence for the existence of a connection between two symptoms, therefore, contributing toward evaluating the potential replicability of network structures (Williams, 2021). Bayesian inference methods enable studying the amount of evidence for null associations and hypothesis testing regarding the strength of associations between different sets of symptoms (Williams, Briganti, Linkowski, & Mulder, 2021; Williams & Mulder, 2020).

The present study employed network analysis to investigate the interrelations between PTSD symptoms, DSO symptoms, trauma-related beliefs, and psychosis symptoms (delusions and auditory hallucinations); using Bayesian methods to test hypotheses within the network. Based on theory and empirical evidence highlighting the role of negative trauma-related beliefs in posttraumatic stress and psychosis among clinical samples (Garety et al., 2001; Hardy et al., 2021; Morrison, Frame, & Larkin, 2003; Panayi et al., 2024), it was predicted that trauma-related beliefs (negative self-concept, negative cognitions about the world, and self-blame) would have the greatest predictability in the network. It was hypothesized that the associations between hallucinations and trauma-related beliefs would be greater than the association between hallucinations and re-experiencing. Lastly, the directionality of the interactions between symptoms was examined to gather further information regarding possible causal mechanisms.

## Method

### Design

The study is a cross-sectional design and utilizes baseline data from the Study of Trauma and Recovery (STAR) (ISRCTN93382525). STAR is a randomized controlled trial evaluating the effectiveness of trauma-focused cognitive behavioral therapy for psychosis among individuals with comorbid PTSD and psychosis. The STAR trial received ethical approval from Camberwell St. Giles Research Ethics Committee (REC 20/LO/0853). Full details of the trial methodology are available in the trial protocol (Peters et al., 2022).

### Participants

Participants (*N* = 305) were recruited from UK outpatient services in England. Inclusion criteria were (i) treatment-seeking adults (≥18 years), (ii) with a PTSD diagnosis according to Diagnostic and Statistical Manual of Mental Disorders 5th edition criteria (American Psychiatric Association, 2013), (iii) a schizophrenia-spectrum diagnosis ascertained using the International Classification of Diseases 10th edition (ICD-10), and (iv) distressing hallucinations and/or delusions (WHO, 1993). Exclusion criteria were (i) current primary diagnosis of substance use disorder, (ii) organic factors implicated in the primary etiology of psychosis and/or PTSD, (iii) currently (or in the previous 3 months) undergoing trauma-focused cognitive behavioral therapy (CBT), (iv) major medication changes within the previous 3 months, (v) insufficient English, and (vi) currently experiencing an acute mental health crisis.

### Measures

#### The International Trauma Questionnaire

The International Trauma Questionnaire (ITQ) assesses for ICD-11 PTSD and cPTSD (Cloitre et al., 2018). Six items assess three PTSD symptom clusters: re-experiencing, avoidance, and sense of threat (two items per cluster). Six items assess three disturbances in self-organization (DSO) symptom clusters; affective dysregulation, negative self-concept, and disturbances in relationships (two items per cluster). Items are scored on a 5-point Likert scale ranging from 0 (‘*not at all*’) to 4 (‘*extremely*’); a score of ≥2 (‘*moderately*’) indicates symptom endorsement. A PTSD diagnosis requires endorsement of one symptom from each PTSD symptom cluster and associated functional impairment. A cPTSD diagnosis requires a PTSD diagnosis plus endorsement of one symptom from each DSO symptom cluster and associated functional impairment. An individual can receive a diagnosis of either PTSD or cPTSD. Total scores for each symptom cluster were used in the current study. The reliability of the PTSD subscale was acceptable (*α* = .72), and the reliability of the DSO subscale was good (*α* = .81).

#### The Psychosis Symptom Rating Scales

The Psychosis Symptom Rating Scales (PSYRATS) is a clinician-administered semistructured interview that measures multidimensional aspects of delusions and auditory hallucinations (Haddock, McCarron, Tarrier, & Faragher, 1999). Eleven items assess auditory hallucinations, and six items assess delusions. Items are rated on a 5-point nominal scale of increasing severity (0–4). For the purposes of the analysis, the frequency factor items for delusions and hallucinations were utilized (Woodward et al., 2014) to examine influence of cPTSD on the occurrence of psychosis, consistent with Hardy et al. (2021). For auditory hallucinations, this included items measuring frequency, duration, and disruption. For delusions, this included items capturing preoccupation amount, preoccupation duration, conviction, and disruption. The reliability of the frequency subscales for delusions (*α* = .9) and hallucinations (*α* = .87) was good.

#### The Posttraumatic Cognitions Inventory

The brief version of the Post-Traumatic Cognitions Inventory (PTCI-9) is a 9-item measure that assesses trauma-related beliefs (Wells et al., 2019). The PTCI-9 comprises three subscales: negative cognitions about the self, negative cognitions about the world, and self-blame. Each subscale is measured by three items, respondents indicate how much they agree with statements on a 7-point Likert scale from 1 (‘*totally disagree*’) to 7 (‘*totally agree*’). The reliability of the subscale for negative cognitions about the self was good (*α* = .78) and for negative cognitions about the world (*α* = .76) and self-blame (*α* = .79) were acceptable.

### Procedure

All measures were self-reported (apart from the PSYRATS which is an interviewer-rated assessment), but researcher administered. Independent assessors obtained informed consent and conducted eligibility and research assessments.

### Analysis

Analyses were conducted in R version 4.3.1 (R Core team, 2023). This study utilized raw data from the STAR trial. Missing data at the item level were handled using the multivariate imputation by chained equation (MICE) package (Zhang, 2016), then centered and scaled. Prior to network estimation, unique variable analysis was conducted using the EGAnet package (Christensen, Garrido, & Golino, 2020). The Bayesian Gaussian graphical model (BGGM) package (Williams & Mulder, 2020) was used to estimate the network structure. The package ‘qgraph’ (Epskamp et al., 2012) was used to visualize the networks. A directed acyclic graph (DAG) was estimated using the ‘bnlearn’ package (Scutari, 2010).

#### Unique variable analysis

Unique variable analysis (UVA) identifies redundant variables, as these can impact accurate and valid network estimation (Christensen, Garrido, & Golino, 2020). Redundant variables are likely to have higher node strength values, due to redundancy rather than increased connectivity to other nodes, which can lead to overestimating the importance of certain nodes (Hallquist, Wright, & Molenaar, 2021).

#### Network estimation

A Gaussian graphical model represents an undirected network of partial correlations, which can be visualized to infer the underlying conditional dependence structure (Højsgaard et al., 2012; Lauritzen, 1996). Networks comprise sets of variables, such as symptoms, depicted by circles called ‘nodes’ and lines called ‘edges’ that visualize the relationships between the variables (Højsgaard et al., 2012; Lauritzen, 1996). If two nodes are connected, they are conditionally dependent (i.e. their partial correlation is nonzero) given all other nodes in the network. Each edge has a weight representing the strength of a partial correlation, and edges can denote a positive or a negative association. PTSD symptoms (re-experiencing, avoidance, and sense of threat), DSO symptoms (affective dysregulation, negative self-concept, and disturbed relationships), trauma-related beliefs (negative cognitions about the self, negative cognitions about the world, and self-blame), and delusions and hallucinations were included as nodes in the network.

A Bayesian Gaussian graphical model was estimated (Williams & Mulder, 2020). Bayesian inference requires a prior probability distribution which generates posterior distributions for the parameters of interest when combined with observed data. A posterior distribution is produced for each partial correlation, enabling the visualization of uncertainty in the estimates (Williams & Mulder, 2020). Network estimation with exploratory hypothesis testing provides an opportunity to assess the supporting evidence for the hypothesis that most accurately predicts the observed data (Williams & Mulder, 2020). A Bayes factor (BF) can be used to index the strength of the evidence for the alternative hypothesis (i.e. the partial correlation is not equal to zero) relative to the null hypothesis (i.e. the partial correlation is equal to zero). The ‘explore’ function was used to determine the conditional (in)dependence structure using ‘exhaustive’ selection, which provides evidence for positive, negative, and null relations. Three network plots are generated to represent these relationships: (i) conditional dependence structure (includes both positive and negative associations), (ii) conditional independence structure, and (iii) and where conditional (in)dependence was ambiguous. The default prior was used (.25), and 5000 samples were drawn from the posterior distribution. To determine the presence of an edge in the network, a BF of 3 was used, which is interpreted as positive evidence in favor of the alternative hypothesis (Kass & Raftery, 1995).

If nodes included in the study hypothesis were not conditionally dependent, the shortest path between two nodes was assessed to determine the strongest indirect connections between nodes (Dijkstra, 1959).

Following network estimation, confirmatory analyses using the ‘confirm’ function in the BGGM package was used to test the preregistered study hypotheses (see aspredicted.org #123136). This allows for testing competing sets of equality constraints on multiple partial correlations (Williams & Mulder, 2020).

In addition, Bayesian *R*^2^ was computed for the nodes in the network, which represents the percentage of variance explained by a node with all other nodes in the network (Williams & Mulder, 2020). Commonly defined as ‘node predictability’ in the network literature (Haslbeck & Fried, 2017), it can be interpreted as how well a node connects to other nodes in the network or the self-determination of the network.

Lastly, to estimate a DAG network, we used the hill-climbing algorithm in the R-package bnlearn (Scutari, 2010). DAG is a dynamic Bayesian network approach that can determine the strength and direction of associations between multiple variables from cross-sectional data (McNally, 2016). We followed guidelines formulated by Briganti, Scutari, and McNally (2023) and bootstrapped 10,000 networks to ensure stability of the results. An optimal cutoff was identified to maximize sensitivity and specificity. To aggregate the results, we computed an averaged network. Edges were considered stable if they appeared in more than 65% of bootstrap networks (screening for existence) and if their direction appeared in more than 50% of networks (screening for orientation). While 85% is the standard default for Gaussian data, we adjusted the stability threshold to 65% to account for the zero-inflated distribution (lower base rates) of psychotic symptoms (e.g. hallucinations). In bootstrap resampling, rarer variables frequently suffer from resampling sparsity in specific subsamples, artificially deflating stability scores. A 65% threshold ensures that directional signals are not discarded simply due to low symptom prevalence (Epskamp, Borsboom, & Fried, 2018). For the included edges, directionality was determined based on the majority of bootstraps but interpreted with caution given the cross-sectional nature of the data, and while they indicate potential directions of association, they cannot establish causality (McNally, 2016).

## Results

### Sample characteristics

The sample characteristics (*N* = 305) are summarized in Table 1. Age ranged from 18 to 65 years with a mean of 38.9 years (*SD* = 12.4) and a median of 38 years; 56.1% of participants identified as women and most participants were white British (73.4%). The most common psychotic disorders were F29 Unspecified nonorganic psychosis (41.6%) and F28 Other nonorganic psychotic disorders (23.9%).

**Table 1.** Sample characteristics Table 1. long description.

	Sample (*N* = 305)
	*n* (*%*)
Gender	
Women	171 (56.1)
Men	127 (41.6)
Nonbinary/Prefer not to say	7 (2.3)
Self-defined ethnicity	
Black African	19 (6.2)
Black Caribbean	13 (4.3)
Mixed ethnicity	19 (6.2)
South Asian	10 (3.3)
White British	224 (73.4)
White other	8 (3.0)
Other	12 (4.0)
Psychotic disorder	
F20 (Schizophrenia)	61 (20.0)
F22 (Persistent delusional disorder)	4 (1.3)
F25 (Schizoaffective disorders)	40 (13.1)
F28 (Other non-organic psychotic disorder)	73 (23.9)
F29 (Unspecified non-organic psychosis)	127 (41.6)
ICD–11 traumatic stress diagnosis	
PTSD	26 (8.6)
Complex PTSD	241 (80.1)

Based on PSYRATS scores, 82% of the sample presented with delusions and 79.3% presented with hallucinations (voices); of these subgroups, the delusion total score was *M =* 17.1 (*SD* = 3.1), and the hallucinations (voices) total score was *M* = 31.4 (*SD* = 5.3).

The frequencies of symptoms included in the network analysis are presented in Table 2. For information on trauma exposure, see the Supplementary Materials (Supplementary Table S3).

**Table 2.** Descriptive statistics of psychosis, trauma-related beliefs, PTSD, and DSO symptoms (*N* = 305) Table 2. long description.

	*M* (*SD*)	Min – max score
Psychosis (*PSYRATS*)		
Delusions (frequency factor score)	8.75 (4.6)	0–15
Hallucinations (frequency factor score)	5.71 (3.57)	0–13
Trauma-related beliefs (*PTCI*)		
Negative cognitions about the self	4.88 (1.54)	0–7
Negative cognitions about the world	5.4 (1.25)	0–7
Self-blame	4.16 (1.84)	0–7
Posttraumatic stress (*ITQ*)		
Re-experiencing	5.45 (2.02)	0–8
Avoidance	6.20 (1.7)	0–8
Sense of threat	5.98 (1.92)	0–8
Disturbances in self-organization (*ITQ*)		
Affective dysregulation	5.97 (1.72)	0–8
Negative self-concept	6.18 (2.27)	0–8
Disturbed relationships	6.18 (1.8)	0–8

*Note:* The study utilized raw baseline data from the STAR trial. Descriptive statistics followed multivariate imputation by chained equation (MICE) to impute missing data at the item level. PSYRATS = The Psychosis Symptom Rating Scales; PTCI = Posttraumatic Cognitions Inventory; ITQ = The International Trauma Questionnaire.

### Unique variable analysis

There was overlap between the PTCI ‘negative cognitions about self’ and ITQ ‘negative sense of self’. The ITQ item was chosen over the PTCI item on conceptual grounds (since we were primarily interested in cPTSD as operationalized in the ITQ) and PTCI ‘negative cognitions about self’ was removed from the analysis.

### Network estimation

**Figure 1.**
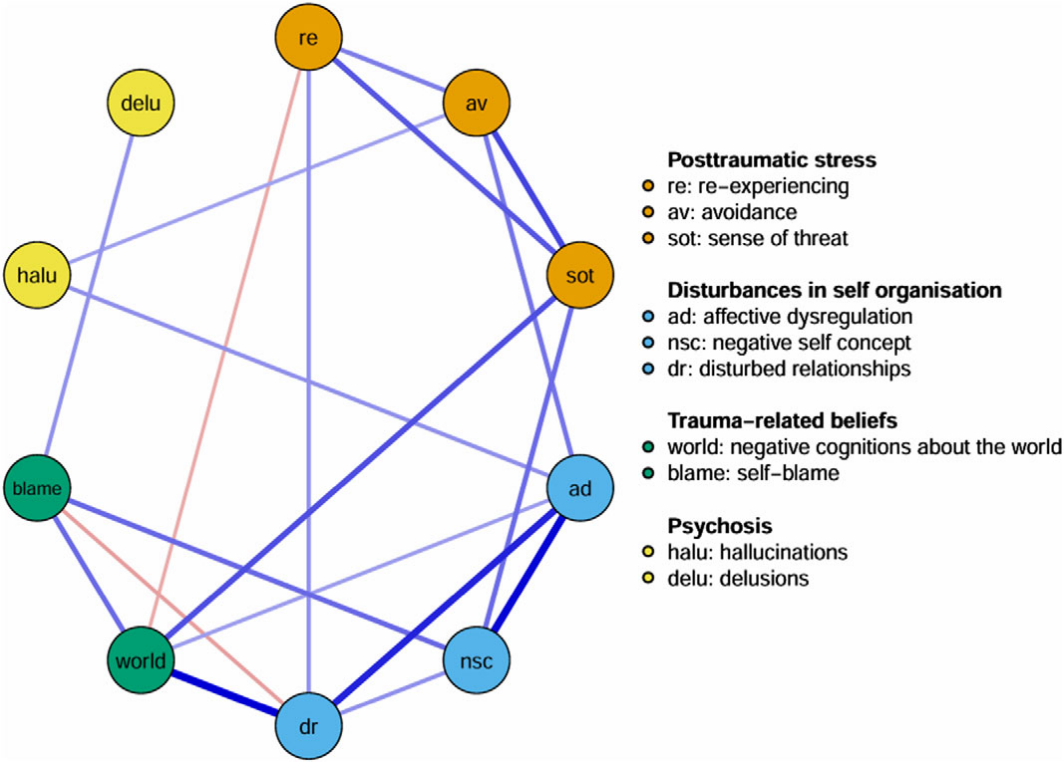
BGGM conditional dependence structure of psychosis symptoms, posttraumatic stress symptoms, disturbances in self-organization symptoms, and trauma-related beliefs. *Note:* Blue lines represent positive associations, and red lines represent negative associations. The color of the node represents the construct to which the symptom belongs. Figure 1. long description.

The conditional dependence network is presented in Figure 1 (for posterior distributions, see Supplementary Figure S1). Hallucinations had a direct connection to affective dysregulation and avoidance. In the conditional independence structure, there was no evidence for connections between hallucinations and self-blame as well as hallucinations and negative cognitions about the world (see Supplementary Figure S2). The evidence for a connection between hallucinations and negative self-concept was ambiguous (see Supplementary Figure S3).

In the conditional dependence structure, delusions had a direct connection to self-blame. Evidence for connections between delusions and negative cognitions about the self as well as delusions and negative cognitions about the world were ambiguous. Notably, there was evidence that hallucinations and delusions were conditionally independent (see Supplementary Figure S2).

The shortest path from (i) hallucinations to re-experiencing was via avoidance; (ii) hallucinations to negative self-concept was via affective dysregulation; (iii) hallucinations to self-blame was via affective dysregulation and negative self-concept; and (iv) hallucinations to negative cognitions about the world was via affective dysregulation and disturbed relationships. The shortest path from (i) delusions to negative cognitions about the world and delusions to negative self-concept was via self-blame and (ii) hallucinations to delusions was via affective dysregulation, negative self-concept, and self-blame.

The preregistered hypothesis specified that the associations between trauma-related beliefs and hallucinations respectively would be greater than the association between re-experiencing and hallucinations. Evidence for the relations specified in the hypothesis was either ambiguous or relations were conditionally independent.

**Figure 2.**
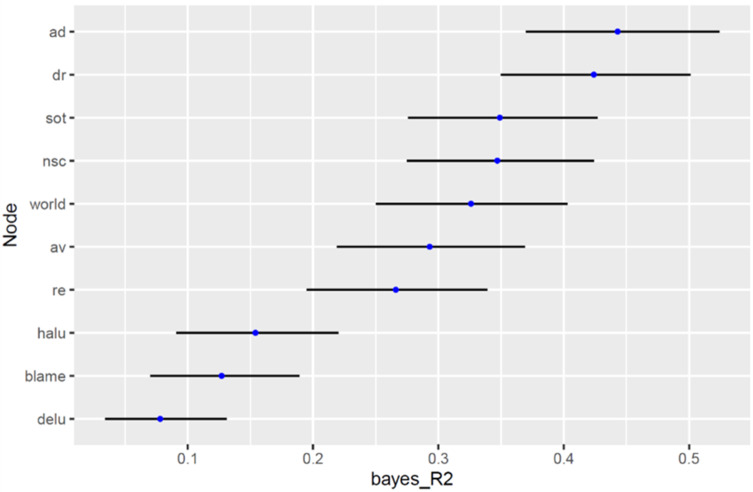
Node predictability (Bayesian *R*^2^) for the 10 nodes in the network. *Note:* Ad = affective dysregulation; halu = hallucinations; av = avoidance; dr = disturbed relationships; blame = self-blame; re = re-experiencing; nsc = negative self-concept; delu = delusions; sot = sense of threat; world = negative cognitions about the world. Figure 2. long description.

Node predictability results are presented in Figure 2. Although affective dysregulation had the highest *R*^2^ score (44.3%), the centrality intervals suggest that the predictability estimates do not significantly differ between nodes; therefore, it is not possible to identify nodes with the greatest predictability in the network, but disturbed relationships and affective dysregulation had relatively higher predictability compared to hallucinations, delusions, and self-blame. Delusions had the lowest *R*^2^ (7.8%).

### Direct acyclic graph

**Figure 3.**
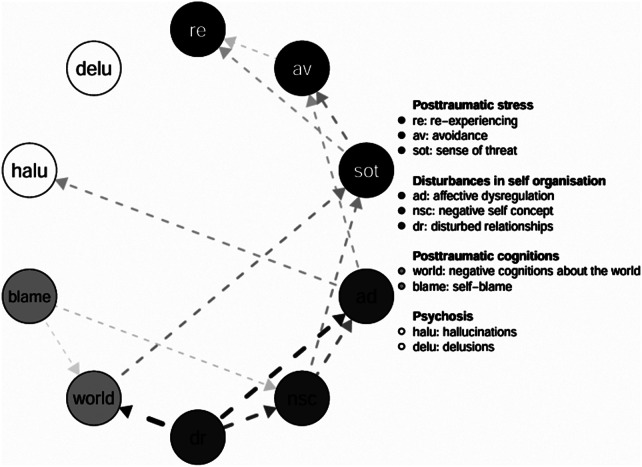
Directed acyclic graph (DAG). *Note:* Edge thickness indicates confidence that the direction of prediction is in the direction depicted in the graph. Figure 3. long description.

In the DAG analysis (see Figure 3), the only node directly predicting hallucinations was affective dysregulation. For findings on strength and directionality, see Supplementary Table S2. Hallucinations and delusions did not directly predict any nodes in the network.

## Conclusions

This study employed network analysis using Bayesian inference to investigate the interrelations between psychosis symptoms and posttraumatic sequelae. To the best of our knowledge, this is one of the first studies to employ standardized assessments to examine the associations between posttraumatic cognitions, psychosis, PTSD and DSO symptoms using network analysis in a sample of people with PTSD and distressing psychosis symptoms. It was predicted that trauma-related beliefs would be among the most central symptoms in the network, but many symptoms had similar predictability scores, and therefore it was not possible to discriminate symptom centrality. Based on previous literature, it was also hypothesized that the association between trauma-related beliefs (negative self-concept, negative cognitions about the world, and self-blame) and hallucinations would be greater compared to the association between re-experiencing and hallucinations. There was no evidence for conditionally dependent relations between re-experiencing and voices, nor between trauma-related beliefs and voices, suggesting no difference between these associations. However, delusions had a positive direct connection with self-blame, while voices had positive direct connections with affective dysregulation (including numbing) and avoidance (including internal suppression). In further analysis of the relationship between symptoms in the network, it was found that affective dysregulation predicted voices, but self-blame did not predict delusions.

There were no direct connections between the ‘core’ re-experiencing symptoms of PTSD and psychosis symptoms when controlling for connections between all nodes. Instead, these symptoms were connected indirectly through other trauma-related mechanisms. Consistent with Hardy et al. (2021) and Panayi et al. (2025), this suggests that the role of episodic memory in maintaining voices and delusions may be relatively less influential compared to other processes, such as emotional regulation and beliefs. Accordingly, we found that avoidance, affective dysregulation, and self-blame had direct associations with psychosis. This supports theory and research proposing that these trauma-related mechanisms play a role in maintaining psychosis symptoms (Berry, Bucci & Varese, 2017; Garety et al., 2001; Hardy, 2017; Longden, Madill, & Waterman, 2012; Morrison, Frame, & Larkin, 2003; Panayi et al., 2024; Steel & Holmes, 2007). Affective dysregulation includes numbing, which is a dissociative phenomenon, and dissociation has been found to mediate the relationship between trauma and voice hearing (Alameda et al., 2020; Longden et al., 2020; Panayi et al., 2022). Dissociative processes interact with sensory-perceptual processing and self-representations in autobiographical memory, which has been hypothesized to shape the occurrence and content of voice hearing (Hardy, 2017). Self-blame may contribute to psychosis, as trauma-related beliefs can negatively shape the content and interpretations of experiences (Hardy et al., 2016; van den Berg et al., 2023). For example, someone believing ‘I am a bad person who deserves to be punished’ might become hypervigilant to signs of danger and misinterpret neutral events as deliberate harm. The same belief can also lead to shame, with the person becoming numb and detached in response to their distress. The resulting dissociation may increase vulnerability to hearing voices due to altered information processing. These findings may have implications for psychological interventions, as targeting these specific posttraumatic stress sequelae could reduce psychosis symptoms. The present study is limited, as numbing was the only item to capture dissociative phenomena. Further research, utilizing standardized validated measures to assess for dissociative symptoms, is required to test these hypotheses.

This study also found that it was not possible to discriminate symptom centrality, in contrast to a previous network analysis study, conducted with a comorbid PTSD and psychosis sample, which reported that trauma-related beliefs were the most central symptoms in the network (Hardy et al., 2021). It is possible that the discrepant findings reflect heterogeneity of the population. There may be subgroups within the PTSD and psychosis population, which may account for the different relationships identified across samples (Borsboom, 2017). For example, in the present sample, voice severity was almost three times higher compared to the severity reported in Hardy et al. (2021), which may have influenced differences in study findings. Identifying subgroups with similar symptom profiles may improve validity and reliability of networks.

This study has several limitations. Given the cross-sectional nature of the study, it is not possible to determine causality between symptoms. Longitudinal studies, such as Panayi et al. (2024), are required to establish temporal relations between symptoms within the network. Therapy trials are underway which will provide further longitudinal data on mechanisms (Burger et al., 2022; Peters et al., 2022). Negative edges were detected in the network that are not consistent with research and theory, the clinical nature of the sample may have introduced Berksons bias, therefore these edges may reflect a statistical artefact (Berkson, 2014; de Ron, Fried, & Epskamp, 2021). Further research is required among clinical and nonclinical populations reflective of the psychosis continuum and continuum of trauma responses. An advantage of Bayesian hypothesis testing for network structure is the ability to evaluate evidence for conditionally (in)dependent associations between symptoms (Williams, 2021). However, many edges remained ambiguous indicating insufficient evidence to determine certain symptom relationships. This ambiguity may stem from limited sample size, sample heterogeneity, or the inherently conservative nature of this estimation approach compared to alternatives (Isvoranu & Epskamp, 2023; Williams, 2021). Future research should also investigate how relationships between PTSD and psychosis are influenced by gender, age, duration of illness, trauma history, post-traumatic stress presentation, and psychosis outcomes.

Targeting key symptoms in a network may offer strategic treatment targets, where ameliorating such symptoms could destabilize the network structure, supporting clinical and functional recovery (Borsboom, 2017; Borsboom & Cramer, 2013; Cramer, Waldorp, van der Maas, & Borsboom, 2010). Given that some trauma-related processes were connected to and predictive of psychosis symptoms, modifying these processes through the use of trauma-focused therapies may improve psychosis symptoms (see Hardy et al., 2024, for an overview). Emerging research demonstrates that trauma-focused therapy is safe and effective for posttraumatic stress symptoms in psychosis and may be beneficial in reducing psychosis symptoms too (Brand, McEnery, Rossell, Bendall, & Thomas, 2018; Hardy, Good, Dix, & Longden, 2022; Keen, Hunter, & Peters, 2017; van den Berg et al., 2015; Varese et al., 2021, 2024).

In conclusion, this study suggests that people’s attempts to regulate the threat associated with trauma, together with the self-blame they ascribe to their traumatic experiences, may play a role in maintaining psychosis. Longitudinal, mechanistic research is required to investigate these hypotheses further. This will inform the development of therapies that are optimally tailored to target the trauma-related psychosocial processes that maintain psychosis. The study adds to evidence highlighting that people with psychosis should be offered trauma assessment, with access to trauma-informed support, including trauma-focused therapies, if indicated (Substance Abuse and Mental Health Services Administration, 2014).

## Supporting information

10.1017/S0033291726104978.sm001 Frost et al. supplementary material Frost et al. supplementary material

## Supplementary material

The supplementary material for this article can be found at http://doi.org/10.1017/S0033291726104978.

## Long descriptions

### Table 1. Long description

The table presents data for a total sample size N equals 305.

Gender distribution:

* Women: 171 (56.1 percent)

* Men: 127 (41.6 percent)

* Nonbinary or Prefer not to say: 7 (2.3 percent)

Self-defined ethnicity:

* Black African: 19 (6.2 percent)

* Black Caribbean: 13 (4.3 percent)

* Mixed ethnicity: 19 (6.2 percent)

* South Asian: 10 (3.3 percent)

* White British: 224 (73.4 percent)

* White other: 8 (3.0 percent)

* Other: 12 (4.0 percent)

Psychotic disorder (I C D codes):

* F 20 (Schizophrenia): 61 (20.0 percent)

* F 22 (Persistent delusional disorder): 4 (1.3 percent)

* F 25 (Schizoaffective disorders): 40 (13.1 percent)

* F 28 (Other non-organic psychotic disorder): 73 (23.9 percent)

* F 29 (Unspecified non-organic psychosis): 127 (41.6 percent)

I C D 11 traumatic stress diagnosis:

* P T S D: 26 (8.6 percent)

* Complex P T S D: 241 (80.1 percent)

Navigate back to Table 1..

### Table 2. Long description

The table consists of three columns: Symptom Category, M (S D), and Min – max score. It is divided into four main sections:

1. Psychosis (P S Y R A T S):

* Delusions (frequency factor score): 8.75 (4.6) with a range of 0–15.

* Hallucinations (frequency factor score): 5.71 (3.57) with a range of 0–13.

2. Trauma-related beliefs (P T C I):

* Negative cognitions about the self: 4.88 (1.54) with a range of 0–7.

* Negative cognitions about the world: 5.4 (1.25) with a range of 0–7.

* Self-blame: 4.16 (1.84) with a range of 0–7.

3. Posttraumatic stress (I T Q):

* Re-experiencing: 5.45 (2.02) with a range of 0–8.

* Avoidance: 6.20 (1.7) with a range of 0–8.

* Sense of threat: 5.98 (1.92) with a range of 0–8.

4. Disturbances in self-organization (I T Q):

* Affective dysregulation: 5.97 (1.72) with a range of 0–8.

* Negative self-concept: 6.18 (2.27) with a range of 0–8.

* Disturbed relationships: 6.18 (1.8) with a range of 0–8.

Navigate back to Table 2..

### Figure 1. Long description

A network diagram with 10 circular nodes arranged in a ring. Blue lines indicate positive associations and red lines indicate negative associations, with line thickness representing strength.

Clockwise from the top:

* re (orange): re-experiencing. Strong blue connections to av and sot.

* av (orange): avoidance. Strong blue connection to sot.

* sot (orange): sense of threat. Strong blue connection to ad.

* ad (light blue): affective dysregulation. Strong blue connection to nsc and dr.

* nsc (light blue): negative self concept. Strong blue connection to dr.

* dr (light blue): disturbed relationships. Strong blue connection to world.

* world (green): negative cognitions about the world. Strong blue connection to blame.

* blame (green): self-blame. Red connection to re.

* halu (yellow): hallucinations. Blue connection to delu.

* delu (yellow): delusions. Blue connection to re.

A legend on the right categorizes the nodes:

* Posttraumatic stress (orange): re, av, sot.

* Disturbances in self organisation (light blue): ad, nsc, dr.

* Trauma-related beliefs (green): world, blame.

* Psychosis (yellow): halu, delu.

Navigate back to Figure 1..

### Figure 2. Long description

The horizontal axis is labeled bayes_R sub 2 with a scale from 0.1 to 0.5. The vertical axis is labeled Node and lists ten categories. From top to bottom, the data points (blue dots) and their horizontal error bars (black lines) are:

* ad (affective dysregulation): dot at 0.44, error bar from 0.37 to 0.52.

* dr (disturbed relationships): dot at 0.42, error bar from 0.35 to 0.50.

* sot (sense of threat): dot at 0.35, error bar from 0.28 to 0.43.

* nsc (negative self-concept): dot at 0.35, error bar from 0.28 to 0.43.

* world (negative cognitions about the world): dot at 0.33, error bar from 0.25 to 0.40.

* av (avoidance): dot at 0.29, error bar from 0.22 to 0.37.

* re (re-experiencing): dot at 0.27, error bar from 0.20 to 0.34.

* halu (hallucinations): dot at 0.15, error bar from 0.09 to 0.22.

* blame (self-blame): dot at 0.13, error bar from 0.07 to 0.19.

* delu (delusions): dot at 0.08, error bar from 0.03 to 0.13.

Navigate back to Figure 2..

### Figure 3. Long description

A circular network diagram with ten nodes connected by dashed arrows of varying thicknesses.

Nodes arranged clockwise from the top-left:

* delu: white circle, no outgoing arrows.

* re: black circle, points to av.

* av: black circle, points to re and sot.

* sot: black circle, points to av, ad, and n s c.

* ad: dark gray circle, points to sot and n s c.

* n s c: dark gray circle, points to ad and d r.

* d r: dark gray circle, points to n s c and world. This arrow to world is the thickest in the diagram.

* world: light gray circle, points to sot.

* blame: light gray circle, points to world.

* halu: white circle, points to world.

Legend on the right categorizes the nodes:

* Posttraumatic stress: re (re-experiencing), av (avoidance), sot (sense of threat).

* Disturbances in self organisation: ad (affective dysregulation), n s c (negative self concept), d r (disturbed relationships).

* Posttraumatic cognitions: world (negative cognitions about the world), blame (self-blame).

* Psychosis: halu (hallucinations), delu (delusions).

Navigate back to Figure 3..
