# Supplementary material for: Symptom networks of therapy-seeking individuals with comorbid psychosis and PTSD
Source: Psychol Med. 2026 Jul 28;56:e239. doi: 10.1017/S0033291726104978 (PMC13439243; doi:10.1017/S0033291726104978)
Supplement: Supplementary file 1 [file S0033291726104978sup001.docx]

## **Table S1. Descriptive statistics for the analysis using imputed data**

*Descriptive statistics of variables included in the network analyses with imputed missing data (N = 305*)

|  | **Mean (SD)** |
| --- | --- |
| **Psychosis** *(PSYRATS)* |  |
| Delusions (frequency factor scores) | 8.78 (4.6) |
| Hallucinations (frequency factor scores) | 5.73 (3.6) |
|  |  |
| **Trauma-related beliefs** *(PTCI)* |  |
| Negative cognitions about the self | 4.86 (1.55) |
| Negative cognitions about the world | 5.40(1.24) |
| Self-blame | 4.16 (1.84) |
|  |  |
| **Posttraumatic stress** *(ITQ)* |  |
| Re-experiencing | 5.4 (2.02) |
| Avoidance | 6.22 (1.69) |
| Sense of threat | 5.97 (1.93) |
|  |  |
| **Disturbances in self-organization** *(ITQ)* |  |
| Affective dysregulation | 6 (1.69) |
| Negative self-concept | 6.20 (2.8) |
| Disturbed relationships | 6.18 (1.8) |

*Note.* PSYRATS = The Psychosis Symptom Rating Scales; PTCI = Posttraumatic Cognitions Inventory; ITQ = The International Trauma Questionnaire.

## **Additional statistical information from the Bayesian network analysis**

**Figure S1**

*Posterior distribution for each partial correlation*

**Figure S2**

*Conditional independence structure*

*Note.* Grey lines provide evidence for the null hypothesis (i.e., partial correlation equals zero, evidence of no association)

**Figure S3**

*Ambiguous network.*

*Note.* Edges represent evidence that was inconclusive.

**Table S2. Directional probabilities of arrows in DAG**

| **Arrows in DAG** | | Strength | Direction |
| --- | --- | --- | --- |
| **From** | **To** |  |  |
| Avoidance | Re-experiencing | 0.7650 | 0.5240523 |
| Sense of threat | Re-experiencing | 0.9719 | 0.5039974 |
| Sense of threat | Avoidance | 0.9719 | 0.5458380 |
| Affective dysregulation | Hallucinations | 0.6819 | 0.7663880 |
| Affective dysregulation | Avoidance | 0.8866 | 0.5512633 |
| Negative self-concept | Affective dysregulation | 0.9979 | 0.6200521 |
| Negative self-concept | Sense of threat | 0.8900 | 0.5930899 |
| Disturbed relationships | Affective dysregulation | 0.9993 | 0.5935655 |
| Disturbed relationships | Negative beliefs world | 0.9995 | 0.6679840 |
| Disturbed relationships | Negative self-concept | 0.6711 | 0.6442408 |
| Negative beliefs world | Sense of threat | 0.9403 | 0.5673189 |
| Self-blame | Negative self-concept | 0.9139 | 0.7489879 |
| Self-blame | Negative beliefs world | 0.8255 | 0.8699576 |

**Table S3. Baseline Characteristics – Trauma Experiences (from Trauma And Life Events (TALE) checklist)**

| **Trauma category** |  |  |  | **TAU** |  |  |  | **Therapy** \| \| |  |  |  | |  | |  | | **Total** | |  |
| --- | --- | --- | --- | --- | --- | --- | --- | --- | --- | --- | --- | --- | --- | --- | --- | --- | --- | --- | --- |
|  |  | **N** | **Overall** | **N** | **Index event(s)** |  | **N** | **Overall** | **N** | **Index event(s)** |  | | **N** | | | **Overall** | | **N** | **Index event(s)** |
| Trauma type (TALE item number)  (n(%)) | 1.War exposure (item 1) | 151 | 7 (4·6%) | 151 | 2 (1·3%) |  | 154 | 11 (7·1%) | 154 | 4 (2·6%) |  | | 305 | | | 18 (5·9%) | | 305 | 6 (2.0%) |
|  | 2.Attachment related (2-4) | 151 | 135 (89·4%) | 151 | 21 (13·9%) |  | 154 | 144 (93·5%) | 154 | 29 (18·8%) |  | | 305 | | | 279 (91·5%) | | 305 | 50 (16.4%) |
|  | 3.Witnessing verbal or physical abuse at home (9) | 151 | 106 (70·2%) | 151 | 17 (11·3%) |  | 154 | 122 (79·2%) | 154 | 14 (9·1%) |  | | 305 | | | 228 (74·8%) | | 305 | 31 (10.2%) |
|  | Any interpersonal (any of below items) | 151 | 150 (99·3%) | 151 | 115 (76·2%) |  | 154 | 153 (99·3%) | 154 | 127 (82·5%) |  | | 305 | | | 303 (99·3%) | | 305 | 242 (79.3%) |
|  | 4. Bullying & discrimination (5+6) | 151 | 132 (87·4%) | 151 | 17 (11·3%) |  | 154 | 141 (91·6%) | 154 | 17 (11·0%) |  | | 305 | | | 273 (89·5%) | | 305 | 34 (11.1%) |
|  | 5. Sexual abuse (13+14) | 150 | 115 (76·7%) | 150 | 69 (46·0%) |  | 153 | 109 (71·2%) | 153 | 75 (49·0%) |  | | 303 | | | 224 (73·9%) | | 303 | 144 (47.5%) |
|  | 6. Emotional abuse (7) | 151 | 136 (90·1%) | 151 | 33 (21·9%) |  | 154 | 130 (84·4%) | 154 | 34 (22·1%) |  | | 305 | | | 266 (87·2%) | | 305 | 67 (22.0%) |
|  | 7. Physical abuse (8+10) | 151 | 139 (92·0%) | 151 | 52 (34·4%) |  | 154 | 141 (91·6%) | 154 | 69 (44·8%) |  | | 305 | | | 280 (91·8%) | | 305 | 121 (39.7%) |
|  | 8. Emotional neglect (11) | 149 | 128 (85·9%) | 149 | 21 (14·1%) |  | 154 | 121 (78·6%) | 154 | 27 (17·5%) |  | | 303 | | | 249 (82·2%) | | 303 | 48 (15.8%) |
|  | 9. Physical neglect (12) | 150 | 54 (36·0%) | 150 | 6 (4·0%) |  | 153 | 66 (43·1%) | 153 | 6 (3·9%) |  | | 303 | | | 120 (39·6%) | | 303 | 12 (4.0%) |
|  | 10. Psychosis-related (15 – 17)* | 151 | 125 (82·8%)* | 151 | 24 (15·9%) |  | 154 | 131 (85·1%)* | 154 | 22 (14·3%) |  | | 305 | | | 256 (83·9%)* | | 305 | 46 (15.1%) |
|  | 11. Criminal justice (18) | 151 | 76 (50·3%) | 151 | 10 (6·6%) |  | 154 | 87 (56·5%) | 154 | 5 (3·2%) |  | | 305 | | | 163 (53·4%) | | 305 | 15 (4.9%) |
|  | 12. Non-interpersonal (19) | 150 | 67 (44·7%) | 150 | 8 (5·3%) |  | 154 | 60 (39·0%) | 154 | 2 (1·3%) |  | | 304 | | | 127 (41·8%) | | 304 | 10 (3.3%) |
|  | 13. Other (20) | 148 | 59 (39·9%) | 148 | 29 (19·6%) |  | 150 | 65 (43·3%) | 150 | 17 (11·3%) |  | | 298 | | | 124 (41·6%) | | 298 | 46 (15.4%) |
| Perceived impact (TALE item 21c) | How much are you affected currently by index event(s) - mean (SD)  (range) | - | - | 142 | 8·65(1·24) (6-10) |  | - | - | 146 | 8·66(1·24)  (5-10) |  | | | - | | - | | 288 | 8.66 (1.24)  (5-10) |
| Multiple exposure (n(%)) | Repeated events* (at least 1 item answered ‘yes’ to ‘more than once’) | 151 | 151(100%) | 150 | 132 (88·0%) |  | 154 | 154 (100%) | 154 | 138 (89·6%) |  | | 305 | | | 305 (100%) | | 304 | 270 (88.8%) |
|  | Multiple trauma types* (more than 1 of above 13 types reported) | 151 | 151(100%) | 151 | 85 (56·3%) |  | 154 | 154 (100%) | 154 | 84 (54·5%) |  | | 305 | | | 305 (100%) | | 305 | 169 (55.4%) |
|  | Number of trauma types* - mean (SD)  (range) | 151 | 8·47 (1·86)  (3 - 12) | 151 | 2·11 (1·56) (1 – 10) |  | 154 | 8·62 (2·15)  (3 - 12) | 154 | 2·17 (1·66) (1 – 12) |  | | 305 | | | 8·55 (2·01)  (3 - 12) | | 305 | 2.14 (1.61) (1 - 12) |
| Trauma timing (n(%)) | Child* (any item <16) | 151 | 142 (94·0%) | 151 | 75 (49·7%) |  | 154 | 145 (94·2%) | 154 | 80 (51·9%) |  | | 305 | | | 287 (94·1%) | | 305 | 155 (50.8%) |
|  | Adult* (any item 16 or over) | 151 | 144 (95·4%) | 151 | 90 (59·6%) |  | 154 | 140 (90·9%) | 154 | 86 (55·8%) |  | | 305 | | | 284 (93·1%) | | 305 | 176 (57.7%) |
|  | Both* (any item < 16 AND 16 or over) | 151 | 134 (88·7%) | 151 | 64 (42·4%) |  | 154 | 127 (82·5%) | 154 | 64 (41·6%) |  | | 305 | | | 261 (85·6%) | | 305 | 128 (42.0%) |
| Index events that meet Criterion A (exposure to actual or threatened death, serious injury, or sexual violence) on CAPS-5 (n [probable or yes] (%)) | | - | - | 146 | 137 (93·8%) |  | - | - | 151 | 140 (92·7%) | |  | - | | | - | | 297 | 277 (93·3) |

*Participants were selected on basis of presence of distressing psychotic experiences (item 15), so this item has been removed as it will, by definition, be endorsed by 100% of the sample. However it has been kept for Index Event(s) to determine the number of participants whose index event(s) was related to their psychotic experiences
